# Links Between Autistic Traits, Feelings of Gender Dysphoria, and Mentalising Ability: Replication and Extension of Previous Findings from the General Population

**DOI:** 10.1007/s10803-020-04626-w

**Published:** 2020-08-01

**Authors:** Aimilia Kallitsounaki, David M. Williams, Sophie E. Lind

**Affiliations:** 1 School of Psychology, Keynes College, University of Kent, Canterbury, CT2 7NP Kent UK; 2 Department of Psychology, City, University of London, Northampton Square, London, EC1V 0HB UK

**Keywords:** Autism, Gender dysphoria, Gender identity, Theory of mind, Mindreading, Replication

## Abstract

Gender nonconformity is substantially elevated in the autistic population, but the reasons for this are currently unclear. In a recent study, Kallitsounaki and Williams (Kallitsounaki and Williams, Journal of Autism and Developmental Disorders, 2020; authors 1 and 2 of the current paper) found significant relations between autistic traits and both gender dysphoric feelings and recalled cross-gender behaviour, and between mentalising ability and gender dysphoric feelings. The current study successfully replicated these findings (results were supplemented with Bayesian analyses), in sample of 126 adults. Furthermore, it extended the previous finding of the role of mentalising in the relation between autistic traits and gender dysphoric feelings, by showing that mentalising fully mediated this link. Results provide a potential partial explanation for the increased rate of gender nonconformity in the autistic population.

**Electronic supplementary material:**

The online version of this article (10.1007/s10803-020-04626-w) contains supplementary material, which is available to authorized users.

## Introduction

In recent years, evidence has accumulated for an overrepresentation of gender nonconformity in autism spectrum disorder (ASD). Gender nonconformity (or gender variance) is an umbrella term used to describe any expression of gender (e.g., identity, behaviour, interests, appearance) that is incongruent with the gender norms of masculinity or femininity (Cooper et al. 2018). Research has shown that autistic adults present significantly more gender dysphoric feelings than people from the general population (George and Stokes 2018), are more likely to express the wish to be the gender opposite to their biological sex/birth-assigned gender (van der Miesen et al. 2018), and to report gender nonconforming identities (Cooper et al. 2018; George and Stokes 2018; Walsh et al. 2018). It is still unclear, however, what underlying neurocognitive mechanisms could explain this phenomenon and few suggestions have been made (for a review, see Van der Miesen et al. 2016).

One potential candidate mechanism/ability that could contribute to gender nonconformity in ASD is the ability to represent mental states, otherwise known as mentalising or theory of mind (Glidden et al. 2016; Jacobs et al. 2014; Van Der Miesen et al. 2016, 2018). While, only initial steps have been made to explore this hypothesis, Kallitsounaki and Williams’ (2020; the first and second authors of the current manuscript) recent findings provided the first evidence in favour of a potential link. Kallitsounaki and Williams investigated the relation between autistic traits (using the Autism-spectrum Quotient; Baron-Cohen et al. 2001b), current gender dysphoric feelings (using the Gender Identity/Gender Dysphoria Questionnaire; Deogracias et al. 2007), and recalled cross-gender behaviour (using the Recalled Childhood Gender Identity/Gender Role Questionnaire; Zucker et al. 2006) among cisgender individuals (i.e., people whose gender identity corresponds with their biological sex/birth-assigned gender). Importantly, Kallitsounaki and Williams also examined the role of mentalising (using the Reading the Mind in the Eyes task; Baron-Cohen et al. 2001a) in the relations between these traits.

Kallitsounaki and Williams (2020) found that the number of self-reported autistic traits was significantly associated with the number of gender dysphoric feelings reported (more autistic traits = more current gender dysphoric feelings). Kallitsounaki and Williams also extended this finding further by reporting a significant association between autistic traits and recalled cross-gender behaviour (more autistic traits = more recalled cross-gender behaviour in childhood) for the first time. Furthermore, to our knowledge this was the first study that observed a large and significant association between mentalising and gender dysphoric feelings (poorer mentalising = more current gender dysphoric feelings). Of equal importance was the finding that mentalising moderated significantly the relation between autistic traits and gender dysphoric feelings. Further analysis showed that this relation was significant when mentalising ability was low, but it was non-significant when mentalising ability was high. Based on these results and on research evidence that suggests ASD is associated with a mentalising deficit (e.g., Brunsdon and Happé 2014; Jones et al. 2018; Kaland et al. 2008; Yirmiya et al. 1998), Kallitsounaki and Williams (2020) concluded that in autistic people “a weakness in the process of mentalising may contribute to increased fluidity of gender identity” (p. 6).

Despite the potential importance of the findings described above, Kallitsounaki and Williams were rightly cautious about drawing strong conclusions, given the preliminary nature of the findings. Arguably, these findings await replication before conclusions can be drawn from—and theory built on the basis of–them with confidence. Replication is of eminent importance considering the crisis of confidence that has affected the discipline of psychology in recent years (Pashler and Wagenmakers 2012). The necessity for conducting replication is now well recognised (Asendorpf et al. 2013; Makel et al. 2012; Pashler and Wagenmakers 2012), and replicability is being characterised as the cornerstone of science (Simons 2014). Furthermore, as Cesario (2014) suggested “researchers themselves need to provide repeated replications of their own work upon initial publication” (p. 41).

On this basis, the first aim of the current study was to attempt a direct replication of Kallitsounaki and Williams’ (2020) recently published findings of the relations between autistic traits, gender dysphoric feelings, recalled cross-gender behaviour, and mentalising ability in a new sample of adults from the general population to increase confidence in the veracity of the original findings. One thing to note is that in the original research, Kallitsounaki and Williams took an individual differences approach to investigate these links. Therefore, that same approach was employed in the current replication study. It is well documented that there are personality characteristics in the neurotypical population that are qualitatively similar to the defining features of ASD, reflecting continuous liability to the disorder throughout the population. Research findings have indicated that autistic traits are normally distributed in the general population (Constantino and Todd 2003; Ronald et al. 2006) and that “unaffected” relatives of autistic people report more autistic traits than people from the general population (Frazier et al. 2014; Pickles et al. 2000; Piven et al. 1994, 1997). As such, the investigation of the relations between autistic traits and other variables can be informative about the nature of ASD itself (e.g., Lind et al. 2020; Nicholson et al. 2018).

The second aim of this study was to extend further the original findings about the moderator role of mentalising in the link between autistic traits and gender dysphoric feelings, by conducting a mediation analysis. As noted previously, Kallitsounaki and Williams (2020) found that the relation between autistic traits and gender dysphoric feelings was particularly pronounced when mentalising ability was low and completely absent when the level of this ability was high. However, the (unexpected and unusual) non-significant association between autistic traits and mentalising ability found in the original study did not allow a mediation model to be tested (e.g., Baron and Kenny 1986).

In support of the original findings, we predicted that the number of self-reported autistic traits would be significantly associated with the number of self-reported current gender dysphoric feelings *and* with the recalled cross-gender behaviour in childhood (more autistic traits = more current gender dysphoric feelings and recalled cross-gender behaviour). We also predicted that mentalising ability would be significantly associated with the number of current gender dysphoric feelings reported (poorer mentalising = more current gender dysphoric feelings). Furthermore, on the basis of previous research evidence (e.g., Baron-Cohen et al. 2001a; Williams et al. 2018), we expected that the number of self-reported autistic traits would be significantly associated with mentalising ability (more autistic traits = poorer mentalising). Lastly, we predicted that mentalising ability would *mediate* the significant relation between autistic traits and current gender dysphoric feelings.

## Method

### Participants

One hundred and twenty-six people (97 female) took part in the current study. The average age of participants was 20.99 (*SD* = 4.10, range 18 to 45) years. English was the first language of 76.2% the sample and two participants reported having a formal diagnosis of ASD. Student participants were rewarded with course credit in partial fulfilment of their degree and people from the general population did not receive any kind of compensation for their participation. All participants gave informed consent and the study was approved by City, University of London’s Psychology Research Ethics Committee. A comparison between the replication and original Kallitsounaki and Williams’ (2020) sample is presented in Supplementary Table 1.

### Materials and Procedure

All the materials described below are identical to the ones employed by Kallitsounaki and Williams (2020). Participants either took part in a laboratory administration of the measures or completed the study online through SONA (i.e., a web-based experiment management system for recruiting and rewarding participants who take part in research studies).^1^

#### Autism-spectrum Quotient

The Autism-spectrum Quotient (AQ; Baron-Cohen et al. 2001b) is a self-report measure of autistic traits. Participants are presented with a series of 50 statements (e.g., “I find social situations easy”) and they are asked to indicate their level of agreement to each of the items, using a 4-point Likert scale that ranges from “definitely agree” to “definitely disagree”. Participants scores range from 0 to 50, with higher scores denoting increased autistic traits.

#### Reading the Mind in the Eyes

The Reading the Mind in the Eyes (RMIE; Baron‐Cohen et al. 2001a) task is a measure that taps mentalising ability. A series of 36 photographs of the eye region of people is presented to participants and their task is to match each of the photographs with the mental state that they represent, choosing one among four different options on each trial. Participants scores range from 0 to 36, with higher scores suggesting better mentalising ability.

#### Gender Identity/Gender Dysphoria Questionnaire for Adolescents and Adults

The Gender Identity/Gender Dysphoria Questionnaire for Adolescents and Adults (GIDYQ-AA; Deogracias et al. 2007) is a self-report measure of gender identity and gender dysphoria. Participants are presented with a series of 27 questions about their feelings, wishes, and thoughts with respect to their gender assigned at birth and their gender identity that they have to answer using a five-point Likert scale that ranges from “always” to “never”. A mean score was calculated for each participant, with lower scores indicating greater gender dysphoria.

#### Recalled Childhood Gender Identity/Gender Role Questionnaire

The Recalled Childhood Gender Identity/Gender Role Questionnaire (RCGI; Zucker et al. 2006) is a 23-item self-report measure that taps upon recalled sex typed behaviour and closeness to parents. In the current study, participants were instructed to respond only to the 18 items that pertain to gender identity and gender role (Zucker et al. 2006), using a 5-point scale. A mean score was calculated for each participant, with lower scores indicating more recalled cross-gender behaviour in childhood.

### Statistical Analysis

Following Kallitsounaki and Williams’ (2020) approach, participants who had a history of ASD (*n* = 2) were not excluded from the analyses described below. To investigate the success of our replication attempt, we employed a series of statistical methods. First, we analysed whether those results reported as significant by Kallitsounaki and Williams (2020) were replicated in the current study. An alpha level of 0.05 was used as the cut-off for statistical significance. In addition, Bayesian analyses were conducted to examine whether the effects found in the original study (Kallitsounaki and Williams 2020) were present or absent in the data from the current study. That is an increasingly used method that estimates the relative strength of the alternative hypothesis over the null, or vice versa (e.g., Dienes 2014). According to Jeffreys’ (1961) criteria, Bayes factors with values larger than 1 indicate increasing evidence for the alternative hypothesis (BF_10_ > 3 = substantial evidence, BF_10_ > 10 = strong evidence, BF_10_ > 30 = very strong evidence, BF_10_ > 100 = decisive evidence), whereas, scores < 1 indicate evidence for the null hypothesis (BF_10_ < 0.33 = substantial evidence, BF_10_ < 0.10 = strong evidence, BF_10_ < 0.03 = very strong evidence, BF_10_ < 0.01 = decisive evidence). Bayesian analyses were performed using JASP 0.8.1.2 (JASP Team 2016). To directly compare the effect sizes of the current replication study with the ones of Kallitsounaki and Williams (2020), a series of Fisher’s *Z* tests was conducted. For the zero order correlations that were carried-out, coefficients *r* are reported as measures of effect size (≥ 0.10 = small effect, ≥ 0.30 = moderate effect, ≥ 0.50 = large effect; Cohen 1992).

To understand the mechanism through which autistic traits were related to current gender dysphoric feelings, a mediation analysis was conducted. Mediation analysis indicates *how* or *by what means* a predictor variable (X) relates to an outcome variable (Y). In other words, mediation describes a situation where a relation between two variables is *explained* by a third variable that is called *mediator* (M) (Field 2013; Preacher and Hayes 2008). The relation between the predictor variable and the outcome variable, partialling out the effect of the mediator is called the *direct effect*, whereas the effect of the predictor on the outcome through the mediator is called the *indirect effect.* The sum of the direct and the indirect effects is the *total effect* (Field 2013; Preacher and Hayes 2008). In our mediation analysis, we examined whether mentalising (M) mediates the relation between autistic traits (X) and gender dysphoric feelings (Y). To test the significance of the indirect effect we employed bootstrapping procedure. That is a resampling technique “from which the sampling distribution of a statistic is estimated by taking repeated samples from the data set” (Field 2013, p. 871). In mediation analyses, this technique is used to compute confidence intervals for the indirect effects. A variable is said to significantly mediate the relation between X and Y, when the indirect effect is significantly different from zero (Preacher and Hayes 2008). In the current study, a mediation analysis was conducted using PROCESS v3.4.1 operated in SPSS, and unstandardized indirect effects were computed for each of 5000 bootstrapped samples.

## Results

Means (*SD*) for performance on the measures used in the current study are presented in Table 1.

**Table 1:** Mean (SD) score on self-report measures and mentalising performance

Variable	*n*	Mean (*SD*)
AQ	126	18.22 (5.89)
RMIE	126	23.06 (5.87)
GIDYQ-AA^a^	125	4.75 (0.44)
RCGI^a^	125	3.91 (0.65)

*AQ* Autism-spectrum Quotient, *RMIE* Reading the Mind in the Eyes task, *GIDYQ-AA* Gender Identity/Gender Dysphoria Questionnaire for Adolescents and Adults, *RCGI* The Recalled Childhood Gender Identity/Gender Role Questionnaire

^a^Due to error, one male participant completed the female version of the GIDYQ-AA and RCGI. Hence, his data has not been included in the analyses

### Association Analyses

A series of Pearson *r* correlations was conducted to investigate the relations between autistic traits (measured with the AQ), mentalising (measured with the RMIE), current gender dysphoric feeling (measured with the GIDYQ-AA), and recalled childhood crossgender behaviour (measured with the RCGI). Table 2 presents the results of the analyses, along with a comparison between the current findings and the results reported in the original study.

**Table 2:** Bivariate correlations differences between the current and the original (Kallitsounaki and Williams 2020) study

Association	Current study	Original study	Fisher’s Z test
AQ × RMIE	*r* = − 0.33***^b^	*r* = − 0.18	*Z* = 1.18, *p* = 0.237
AQ × GIDYQ-AA	*r* = − 0.31***^a^	*r* = − 0.32**	*Z* = − 0.07, *p* = 0.948
AQ × RCGI	*r* = − 0.34***^b^	*r* = − 0.33 **	*Z* = 0.10, *p* = 0.921
RMIE × GIDYQ-AA	*r* = 0.55***^b^	*r* = 0.70***	*Z* = − 1.91, *p* = 0.057

**p < .01, ***p < .001

*AQ* Autism-spectrum Quotient, *RMIE* Reading the Mind in the Eyes task, *GIDYQ-AA* Gender Identity/Gender Dysphoria Questionnaire for Adolescents and Adults, *RCGI* The Recalled Childhood Gender Identity/Gender Role Questionnaire

^a^BF_10_ = 30–99 (very strong evidence for alternative hypothesis). ^b^BF_10_ ≥ 100 (decisive evidence for alternative hypothesis)

In keeping with predictions, AQ score was moderately, negatively, and significantly associated with both GIDYQ-AA and RCGI. That is, the more autistic traits a person self-reported, the more current gender dysphoric feelings *and* recalled cross-gender behaviour they reported. Bayes factors indicated very strong and decisive evidence in support of the alternative hypotheses, respectively. Fisher’s *Z* tests revealed that neither the AQ $\times$ GIDYQ-AA nor the AQ $\times$ RCGI correlations differed significantly from the equivalent correlations reported in the original study. AQ score was also found to be moderately, negatively and significantly associated with performance on the RMIE task, suggesting that the greater a person’s self-reported autistic traits the lower their mentalising ability. Bayes factors indicated decisive evidence in support of the alternative hypothesis. Furthermore, as predicted, the RMIE $\times$ GIDYQ-AA correlation was positive, large, and significant, just as it was in the original study, even though the size of the two associations was marginally significantly different (somewhat stronger in the original study).^2^ Results suggest that the higher the score on the RMIE, the fewer the current gender dysphoric feelings, and Bayesian analysis indicated decisive evidence in support of the alternative hypothesis.

### Mediation Analysis

**Fig. 1:**
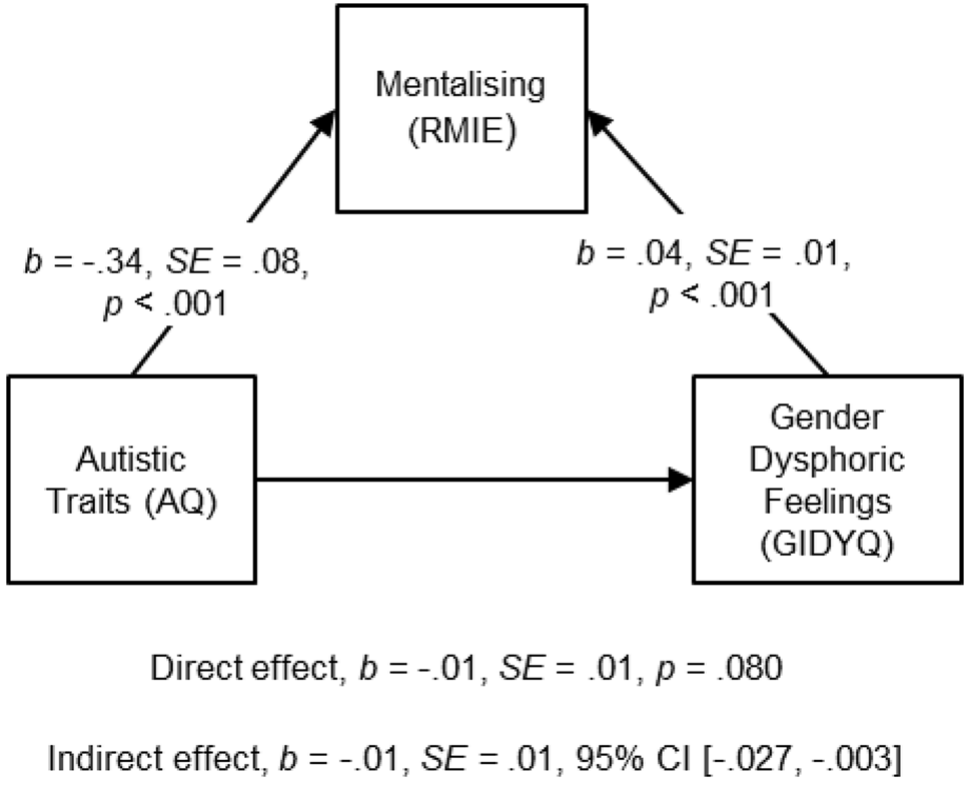
Model of autistic traits as predictor of gender dysphoric feelings, mediated by mentalising ability. *AQ* autism-spectrum quotient, *RMIE* reading the mind in the eyes task, *GIDYQ* Gender Identity/Gender Dysphoria Questionnaire for Adolescents and Adults

A mediation analysis was carried out to examine whether mentalising mediated the effect of autistic traits on current gender dysphoric feelings (see Fig. 1).

As Fig. 1 illustrates, the indirect effect was significantly different from zero, indicating that mentalising mediated the significant relation between autistic traits and current gender dysphoric feelings. While the total effect between autistic traits and gender dysphoric feelings was negative and significant, *b* = − 0.02, *SE* = 0.01, *p* < 0.001, the direct effect between these variables was non-significant. Importantly, results indicate that mentalising ability accounted for most of the relation between autistic traits and current gender dysphoric feelings.

## Discussion

The first aim of the current study was to replicate the study conducted by Kallitsounaki and Williams (2020; first and second authors of the current manuscript). Our replication attempt yielded support for the hypothesis that autistic traits are significantly associated with both current gender dysphoric feelings and recalled cross-gender behaviour. In particular, we found that the more autistic traits a person self-reported, the more gender dysphoric feelings they had in the last 12 months and the more cross-gender behaviour they recalled from childhood. The significant association between autistic traits and gender dysphoric feelings observed in both the original *and* the current study was also consistent with George and Stokes’ (2018) findings. As such, the link between autistic traits and gender dysphoria in the general population appears robust and reliable. Furthermore, our successful replication of the relation between autistic traits and recalled childhood cross-gender behaviour fits well with previous research evidence that autistic children show a weaker preference for sex-typical play than typically developing children (Knickmeyer et al. 2008). In keeping with the original research evidence, we also observed a significant association between mentalising ability and current gender dysphoric feelings. This supports the hypothesis that poorer mentalising ability relates to more gender dysphoric feelings. Given that the link between mentalising and gender dysphoric feelings was reported for first time by Kallitsounaki and Williams (2020), the replication of this link in the current research is striking and increases confidence in the reliability of the original findings.

The second aim of the current study was to extend Kallitsounaki and Williams’ (2020) findings about the role of mentalising in the link between autistic traits and gender dysphoric feelings, examining whether mentalising mediates this link. In contrast to previous research evidence (e.g., Baron-Cohen et al. 2001a; Williams et al. 2018), Kallitsounaki and Williams observed a non-significant association between autistic traits and mentalising. However, in the current study, the number of self-reported autistic traits was moderately, negatively and significantly associated with mentalising ability and therefore, we were able to test our mediation hypothesis. Results from a mediation analysis revealed that the relation between autistic traits and gender dysphoric feelings in the general population was almost entirely explained by mentalising.

Taken together, the replicated and novel findings of the current study suggest it is appropriate to draw a series of conclusions. First, results imply that people with low mentalising ability are more liable than people with high mentalising ability to experience gender dysphoric feelings, and that people with high autistic traits report increased gender dysphoric feelings mainly because their mentalising ability is low. ASD is known to be characterised by diminished mentalising (e.g., Yirmiya et al. 1998), with some suggesting that this represents a cognitive marker of ASD (e.g., Brunsdon and Happé 2014), and research has shown that autistic people experience significantly more gender dysphoric feelings than neurotypical people. They also show a more diverse range of gender identities (George and Stokes 2018), and are more likely to begin or plan to begin the process of gender transitioning (Cooper et al. 2018). As such, it is plausible to suggest that the results of the current study support the hypothesis that mentalising contributes to the overrepresentation of gender nonconformity in ASD (Glidden et al. 2016; Jacobs et al. 2014; Van Der Miesen et al. 2016, 2018).

From a theoretical perspective, a mentalising deficit could contribute to gender nonconformity in a number of ways. Gender constancy—the understanding that one’s own sex does not change regardless of changes in gender-typed appearance, activities, and traits—is considered one of the major cognitive stages that a child needs to reach to be able to formulate a gender identity (e.g., Kohlberg 1966). Indeed, a developmental lag in the acquisition of gender constancy has been found in children with gender identity difficulties (Zucker et al. 1999). Crucially, the level of understanding of gender constancy is related to the ability to distinguish between appearance and reality (Trautner et al. 2003; Zmyj and Bischof-Köhler 2015), which is one of the key components of mentalising. As such, it could be argued that an autistic child who has not reached an adequate level of understanding of gender constancy, due to difficulties in mentalising, could be susceptible to increased cross-gender behaviour in childhood and therefore greater likelihood of developing feelings of gender dysphoria or gender nonconformity in adolescence and adulthood (e.g., Drummond et al. 2008; Green 1987; Wallien and Cohen-Kettenis 2008).

Furthermore, people tend to conform to social conditioning and social norms partially to avoid feelings of guilt and embarrassment that result from others’ judgments when they do not conform (e.g., Scheff 1988; Suhay 2015). The experience of these feelings, known as self-conscious emotions, is thought to depend on people’s mentalising ability (Hobson et al. 2006). A person who experiences difficulties in attributing mental states to others will show a reduced propensity to experience self-conscious emotions and, therefore, might be less likely to comply with societal norms. Indeed, numerous studies have shown that people with ASD experience self-conscious emotions less frequently than neurotypical people (e.g., Capps et al. 1992; Davidson et al. 2018; Losh and Capps 2006). Arguably, if autistic people tend to feel fewer self-conscious emotions, they may be less affected by what other people think about their cross-gender behaviour and therefore less likely to feel pressure to conform of gender norms.

In sum, the current study successfully reproduced (a) the link between autistic traits and current gender dysphoric feelings/recalled cross-gender behaviour and (b) the relation between mentalising and current gender dysphoric feelings, reported by Kallitsounaki and Williams (2020). Most importantly, we found for the first time that the relation between autistic traits and gender dysphoric feelings was to a significant degree explained by mentalising ability. Although direct replication by other laboratories awaits, the main implication of these findings is that mentalising ability could be one of the underling neurocognitive mechanisms that explain the increased prevalence of gender nonconformity in people with a diagnosis of ASD. This represents a highly novel contribution to the literature and provides motivation for future theory-building research on the role of mentalising in the formation of typical and atypical gender self-concepts.

## Electronic supplementary material

Below is the link to the electronic supplementary material.

Supplementary file1 (DOCX 18 kb)

## Footnotes

1 We should note that the data collection was conducted by six trained psychology undergraduate students at City, University of London. The authors of the original paper (i.e., Aimilia Kallitsounaki and David Williams) had no involvement in the process of data collection – this was supervised by Sophie Lind (third author).

2 It should be noted that when participants who reported possession of an ASD diagnosis were removed from the sample of the original study the RMIE $\times$ GIDYQ-AA correlation was almost identical (i.e., *r* = .59) to the one reported in the current study that contained only two participants who had a history of ASD.

## References

[CR1] Asendorpf JB, Conner M, De Fruyt F, De Houwer J, Denissen JJ, Fiedler K, Perugini M (2013). Recommendations for increasing replicability in psychology. European Journal of Personality.

[CR2] Baron RM, Kenny DA (1986). The moderator–mediator variable distinction in social psychological research: Conceptual, strategic, and statistical considerations. Journal of Personality and Social Psychology.

[CR3] Baron-Cohen S, Wheelwright S, Hill J, Raste Y, Plumb I (2001). The “Reading the Mind in the Eyes” test revised version: A study with normal adults, and adults with Asperger syndrome or high-functioning autism. Journal of Child Psychology and Psychiatry.

[CR4] Baron-Cohen S, Wheelwright S, Skinner R, Martin J, Clubley E (2001). The autism-spectrum quotient (AQ): Evidence from Asperger syndrome/high-functioning autism, males and females, scientists and mathematicians. Journal of Autism and Developmental Disorders.

[CR5] Brunsdon VE, Happé F (2014). Exploring the ‘fractionation’ of autism at the cognitive level. Autism.

[CR6] Capps L, Yirmiya N, Sigman M (1992). Understanding of simple and complex emotions in non-retarded children with autism. Journal of Child Psychology and Psychiatry.

[CR7] Cesario J (2014). Priming, replication, and the hardest science. Perspectives on Psychological Science.

[CR8] Cohen J (1992). Statistical power analysis. Current Directions in Psychological Science.

[CR9] Constantino JN, Todd RD (2003). Autistic traits in the general population: A twin study. Archives of General Psychiatry.

[CR10] Cooper K, Smith LG, Russell AJ (2018). Gender identity in autism: Sex differences in social affiliation with gender groups. Journal of Autism and Developmental Disorders.

[CR11] Davidson D, Hilvert E, Misiunaite I, Giordano M (2018). Proneness to guilt, shame, and pride in children with Autism Spectrum Disorders and neurotypical children. Autism Research.

[CR12] Deogracias JJ, Johnson LL, Meyer-Bahlburg HF, Kessler SJ, Schober JM, Zucker KJ (2007). The gender identity/gender dysphoria questionnaire for adolescents and adults. Journal of Sex Research.

[CR13] Dienes Z (2014). Using Bayes to get the most out of non-significant results. Frontiers in Psychology.

[CR14] Drummond KD, Bradley SJ, Peterson-Badali M, Zucker KJ (2008). A follow-up study of girls with gender identity disorder. Developmental Psychology.

[CR15] Field A (2013). Discovering statistics using IBM SPSS statistics.

[CR16] Frazier TW, Ratliff KR, Gruber C, Zhang Y, Law PA, Constantino JN (2014). Confirmatory factor analytic structure and measurement invariance of quantitative autistic traits measured by the social responsiveness scale 2. Autism.

[CR17] George R, Stokes MA (2018). Gender identity and sexual orientation in autism spectrum disorder. Autism.

[CR18] Glidden D, Bouman WP, Jones BA, Arcelus J (2016). Gender dysphoria and autism spectrum disorder: A systematic review of the literature. Sexual Medicine Reviews.

[CR19] Green R (1987). The "sissy boy syndrome" and the development of homosexuality.

[CR20] Hobson RP, Chidambi G, Lee A, Meyer J (2006). Foundations for self-awareness: An exploration through autism. Monographs of the Society for Research in Child Development.

[CR21] Jacobs LA, Rachlin K, Erickson-Schroth L, Janssen A (2014). Gender dysphoria and co-occurring autism spectrum disorders: review, case examples, and treatment considerations. LGBT Health.

[CR22] JASP Team (2016). JASP (Version 0.8) [Computer software].

[CR23] Jeffreys H (1961). Theory of probability.

[CR24] Jones CR, Simonoff E, Baird G, Pickles A, Marsden AJ, Tregay J, Charman T (2018). The association between theory of mind, executive function, and the symptoms of autism spectrum disorder. Autism Research.

[CR25] Kaland N, Callesen K, Møller-Nielsen A, Mortensen EL, Smith L (2008). Performance of children and adolescents with Asperger syndrome or high-functioning autism on advanced theory of mind tasks. Journal of Autism and Developmental Disorders.

[CR26] Kallitsounaki A, Williams D (2020). Mentalising moderates the link between autism traits and current gender dysphoric features in primarily non-autistic, cisgender individuals. Journal of Autism and Developmental Disorders.

[CR27] Knickmeyer RC, Wheelwright S, Baron-Cohen SB (2008). Sex-typical play: Masculinization/defeminization in girls with an autism spectrum condition. Journal of Autism and Developmental Disorders.

[CR28] Kohlberg LA, Maccoby EC (1966). A cognitive developmental analysis of children’s sex role concepts and attitudes. The development of sex differences.

[CR29] Lind SE, Williams DM, Nicholson T, Grainger C, Carruthers P (2020). The self-reference effect on memory is not diminished in autism: Three studies of incidental and explicit self-referential recognition memory in autistic and neurotypical adults and adolescents. Journal of Abnormal Psychology.

[CR30] Losh M, Capps L (2006). Understanding of emotional experience in autism: Insights from the personal accounts of high-functioning children with autism. Developmental Psychology.

[CR31] Makel MC, Plucker JA, Hegarty B (2012). Replications in psychology research: How often do they really occur?. Perspectives on Psychological Science.

[CR32] Nicholson TM, Williams DM, Grainger C, Christensen JF, Calvo-Merino B, Gaigg SB (2018). Interoceptive impairments do not lie at the heart of autism or alexithymia. Journal of Abnormal Psychology.

[CR33] Pashler H, Wagenmakers EJ (2012). Editors’ introduction to the special section on replicability in psychological science: A crisis of confidence?. Perspectives on Psychological Science.

[CR34] Pickles A, Starr E, Kazak S, Bolton P, Papanikolaou K, Bailey A, Rutter M (2000). Variable expression of the autism broader phenotype: Findings from extended pedigrees. The Journal of Child Psychology and Psychiatry and Allied Disciplines.

[CR35] Piven J, Palmer P, Landa R, Santangelo S, Jacobi D, Childress D (1997). Personality and language characteristics in parents from multiple-incidence autism families. American Journal of Medical Genetics.

[CR36] Piven J, Wzorek M, Landa R, Lainhart J, Bolton P, Chase GA, Folstein S (1994). Personality characteristics of the parents of autistic individuals. Psychological Medicine.

[CR37] Preacher KJ, Hayes AF (2008). Asymptotic and resampling strategies for assessing and comparing indirect effects in multiple mediator models. Behavior Research Methods.

[CR38] Ronald A, Happé F, Price TS, Baron-Cohen S, Plomin R (2006). Phenotypic and genetic overlap between autistic traits at the extremes of the general population. Journal of the American Academy of Child & Adolescent Psychiatry.

[CR39] Scheff TJ (1988). Shame and conformity: The deference-emotion system. American Sociological Review.

[CR40] Simons DJ (2014). The value of direct replication. Perspectives on Psychological Science.

[CR41] Suhay E (2015). Explaining group influence: The role of identity and emotion in political conformity and polarization. Political Behavior.

[CR42] Trautner HM, Gervai J, Németh R (2003). Appearance–reality distinction and development of gender constancy understanding in children. International Journal of Behavioral Development.

[CR43] van der Miesen AI, Hurley H, Bal AM, de Vries AL (2018). Prevalence of the wish to be of the opposite gender in adolescents and adults with autism spectrum disorder. Archives of Sexual Behavior.

[CR44] Van Der Miesen AI, Hurley H, De Vries AL (2016). Gender dysphoria and autism spectrum disorder: A narrative review. International Review of Psychiatry.

[CR45] Wallien MS, Cohen-Kettenis PT (2008). Psychosexual outcome of gender-dysphoric children. Journal of the American Academy of Child & Adolescent Psychiatry.

[CR46] Walsh RJ, Krabbendam L, Dewinter J, Begeer S (2018). Brief report: Gender identity differences in autistic adults: Associations with perceptual and socio-cognitive profiles. Journal of Autism and Developmental Disorders.

[CR47] Williams DM, Bergström Z, Grainger C (2018). Metacognitive monitoring and the hypercorrection effect in autism and the general population: Relation to autism (-like) traits and mindreading. Autism.

[CR48] Yirmiya N, Erel O, Shaked M, Solomonica-Levi D (1998). Meta-analyses comparing theory of mind abilities of individuals with autism, individuals with mental retardation, and normally developing individuals. Psychological Bulletin.

[CR49] Zmyj N, Bischof-Köhler D (2015). The development of gender constancy in early childhood and its relation to time comprehension and false-belief understanding. Journal of Cognition and Development.

[CR50] Zucker KJ, Bradley SJ, Kuksis M, Pecore K, Birkenfeld-Adams A, Doering RW, Wild J (1999). Gender constancy judgments in children with gender identity disorder: Evidence for a developmental lag. Archives of Sexual Behavior.

[CR51] Zucker KJ, Mitchell JN, Bradley SJ, Tkachuk J, Cantor JM, Allin SM (2006). The recalled childhood gender identity/gender role questionnaire: Psychometric Properties. Sex Roles.

