# Supplementary material for: Links Between Autistic Traits, Feelings of Gender Dysphoria, and Mentalising Ability: Replication and Extension of Previous Findings from the General Population
Source: J Autism Dev Disord. 2020 Aug 1;51(5):1458–65. doi: 10.1007/s10803-020-04626-w (PMC8084764; doi:10.1007/s10803-020-04626-w)
Supplement: Supplementary file 1 — Supplementary file1 (DOCX 18 kb) [file 10803_2020_4626_MOESM1_ESM.docx]

**Supplementary Material**

1. **A comparison between the replication and the original sample (Kallitsounaki and Williams 2020).**

| Supplementary Table 1 | | | |
| --- | --- | --- | --- |
| *Comparison between Replication and Original Sample* | | | |
| Characteristic | Current study  (*N* = 126) | Original study  (*N* = 101) | Comparison |
| Age in years | *M* = 20.99  (*SD* = 4.10) | *M* = 36.93  (*SD* = 10.11) | *t*(126.38) = -14.89, *p* < .001 |
| Birth-assigned gender | Male: 23%  Female: 77% | Male: 50.50%  Female: 49.50% | χ^2^ = 18.55, *p* < .001 |
| Native English speakers | 76.20% | 94.10% | χ^2^ = 13.42, *p* <.001 |
| Autistic participants | 1.60% | 12.90% | χ^2^ = 11.57, *p* = .001 |
|  | | | |
